# Supplementary material for: Ubiquitination of phytoene synthase 1 precursor modulates carotenoid biosynthesis in tomato
Source: Commun Biol. 2020 Dec 3;3:730. doi: 10.1038/s42003-020-01474-3 (PMC7713427; doi:10.1038/s42003-020-01474-3)
Supplement: Supplementary file 1 — Supplementary Information [file 42003_2020_1474_MOESM1_ESM.pdf]

Ubiquitination of phytoene synthase 1 precursor modulates carotenoid biosynthesis in tomato

Supplementary Information

Peiwen Wang, Yuying Wang, Weihao Wang, Tong Chen, Shiping Tian, Guozheng Qin

Contents

Supplementary Figure 1…..2

Supplementary Figure 2…..3

Supplementary Figure 3…..4

Supplementary Figure 4…..5

Supplementary Figure 5…..6

Supplementary Figure 6…..7

Supplementary Figure 7…..8

Supplementary Figure 8…..9

Supplementary Figure 9…..10

Supplementary Figure 10…...12

Supplementary Figure 11…...13

Supplementary Figure 1

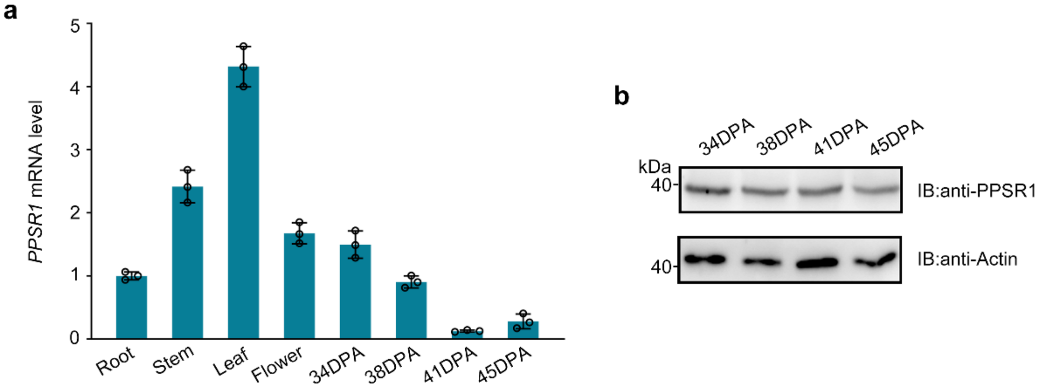

Supplementary Figure 1. Gene expression pattern of *PPSR1* in tomato.

a, The mRNA levels of *PPSR1* in vegetative and reproductive organs of tomato. Total RNA was isolated from root, stem, leaf, flower, and fruits at 34, 38, 41, and 45 days post-anthesis (DPA). The gene transcript levels were measured by quantitative real-time PCR. The *ACTIN* gene was used as the internal control. Error bars represent the means ± standard deviation (SD) of three independent experiments. The circles indicate individual data points. **b**, The protein levels of PPSR1 in tomato fruits during ripening. Total proteins were extracted from the wild-type fruits and detected by immunoblot using an anti-PPSR1 antibody. The actin protein concentration was used as the loading control.

Supplementary Figure 2

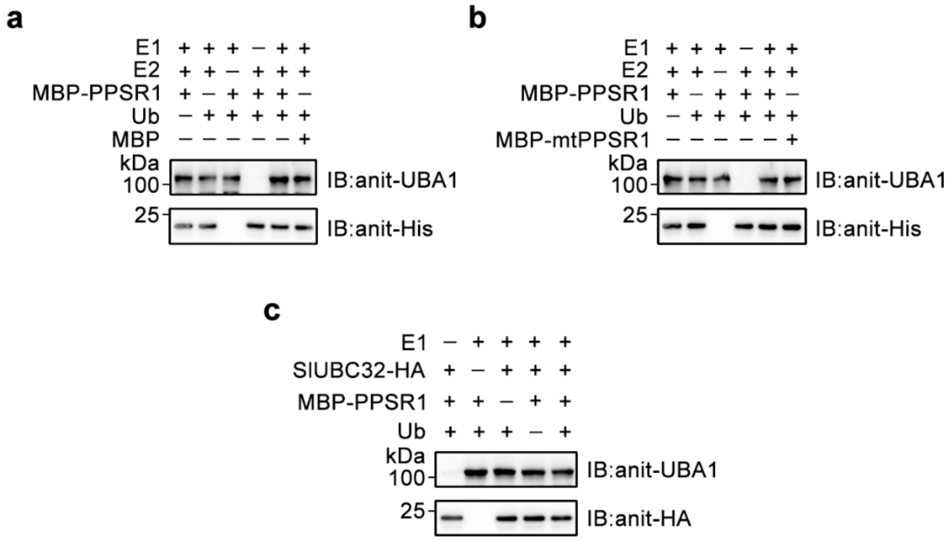

Supplementary Figure 2. The loading control of E1, E2, and SlUBC32-HA in the *in vitro* ubiquitination assays.

a and **b**, The loading control of E1 and E2 in the *in vitro* ubiquitination reactions of MBP-PPSR1 (**a**) and MBP-mtPPSR1 (**b**). The equal loading of E1 and E2 was confirmed by immunoblot analysis using anti-UBA1 and anti-His antibodies, respectively. **c**, The loading control of SlUBC32-HA in the *in vitro* ubiquitination reactions of MBP-PPSR1. The equal loading of SlUBC32-HA was confirmed by immunoblot analysis using an anti-HA antibody. IB, immunoblot.

Supplementary Figure 3

Target site	Off-target sequence	Off-score	Off-target locus	Region	Off-target
T3-OFF1	TTAAGACTAACTCATAACCTAGG	0.243	SL2.50ch10:-18186986	Intergenic	No
T3-OFF2	CTCATACACACTCATACCCTTGG	0.144	SL2.50ch08:+17744368	Intergenic	No
T3-OFF3	TTCAGTCTCACTAATACTCTTGG	0.124	SL2.50ch04:+17047293	Intergenic	No
T3-OFF4	TTCACACTCATTCATACCTATGG	0.105	SL2.50ch07:-47217307	Intergenic	No
T4-OFF1	AGAAATGAGTCATCTTCCCAAGG	0.136	SL2.50ch07:+19758950	Intergenic	No
T4-OFF2	AGAAATGGTCCACCTCCCCTCGG	0.119	SL2.50ch06:+5961146	Intergenic	No

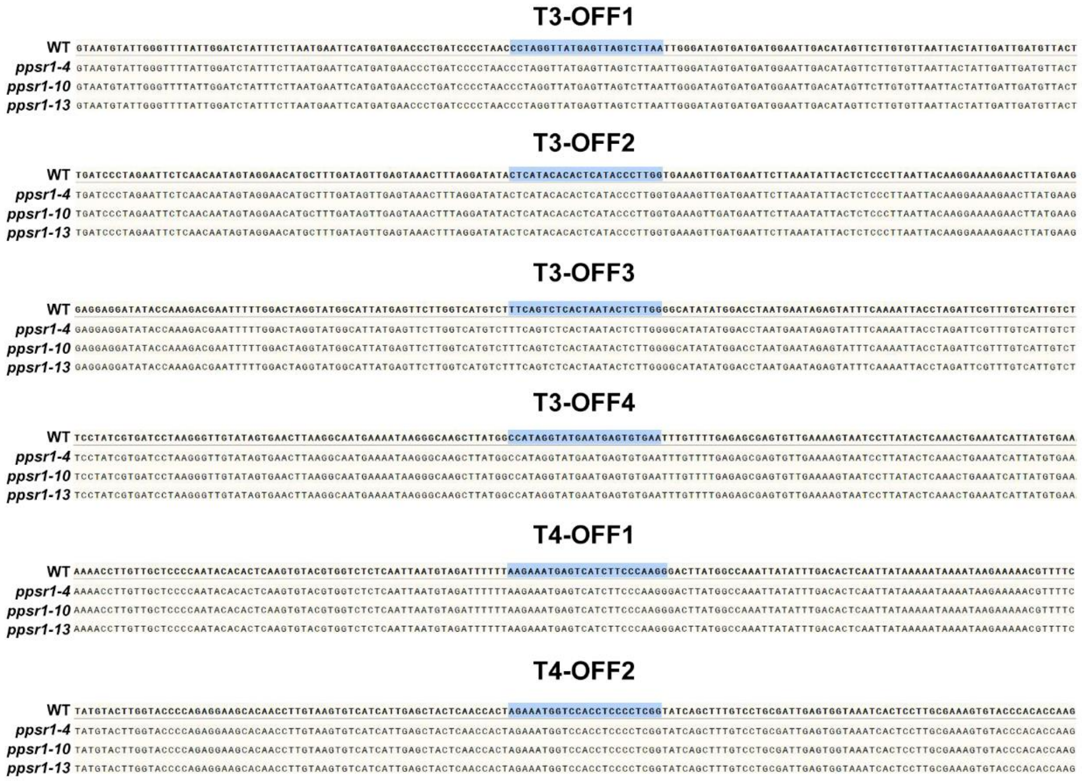

Supplementary Figure 3. Off-target analysis in *ppsr1-4*, *ppsr1-10*, and *ppsr1-13* mutants.

Potential off-target sites were predicted by CRISPR-P (version 2.0, http://crispr.hzau.edu.cn/CRISPR2/). Red letters indicate the protospacer adjacent motif (PAM). The potential off-target sites were detected by direct sequencing of PCR products from genomic DNA flanking these sites. Sequence alignment of 150-bp nucleotides around the potential off-target sites between wild-type (WT) and *ppsr1* mutants is shown. The potential off-targets are highlighted with blue background.

Supplementary Figure 4

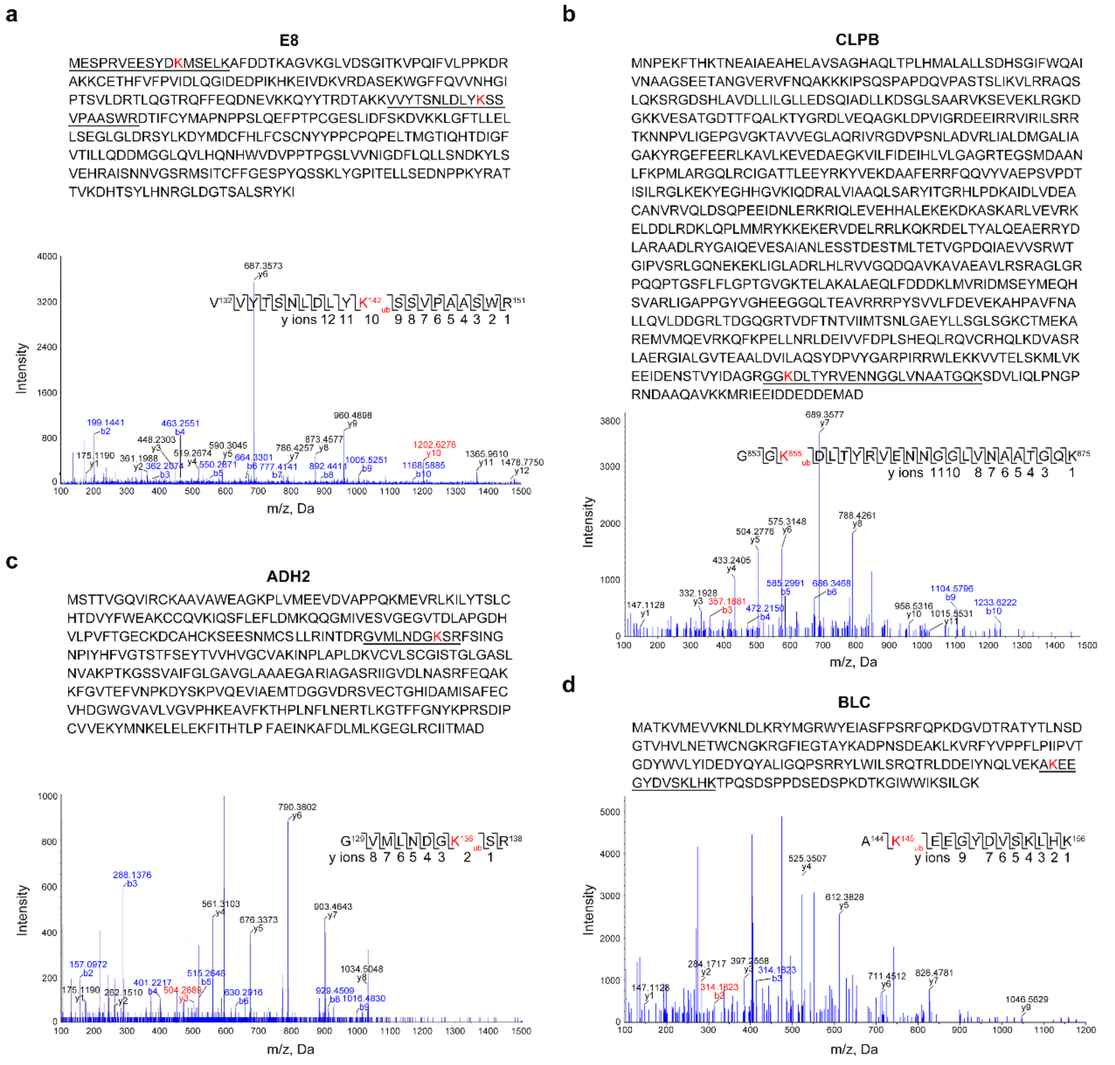

Supplementary Figure 4. Identification of ubiquitination sites in E8, CLPB, ADH2, and BLC by nanoLC–MS/MS.

Sequences of the identified ubiquitinated peptides are underlined in the protein sequences of E8 (**a**), CLPB (**b**), ADH2 (**c**), and BLC (**d**), respectively. The mass spectra of the representative ubiquitinated peptides are displayed. The y-ions and the corresponding peptide sequence are presented, with ubiquitinated lysine (K) residue marked in red. E8, 1-aminocyclopropane-1-carboxylate oxidase-like protein; CLPB, ClpB chaperone; ADH2, alcohol dehydrogenase 2; BLC, outer membrane lipoprotein blc.

Supplementary Figure 5

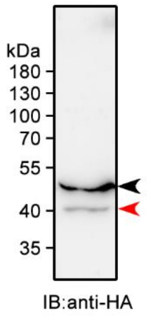

Supplementary Figure 5. Detection of mature PSY1-HA in tobacco (*Nicotiana benthamiana*).

Total proteins were extracted from tobacco (*Nicotiana benthamiana*) leaves expressing PSY1-HA fusion protein by a phenol extraction method and submitted for immunoblot assay using an anti-HA antibody. The full-length PSY1-HA fusion protein has a predicted molecular mass of ~48-kDa (indicated by black arrowhead), and the mature PSY1-HA has a predicted molecular mass of ~41-kDa (indicated by red arrowhead).

Supplementary Figure 6

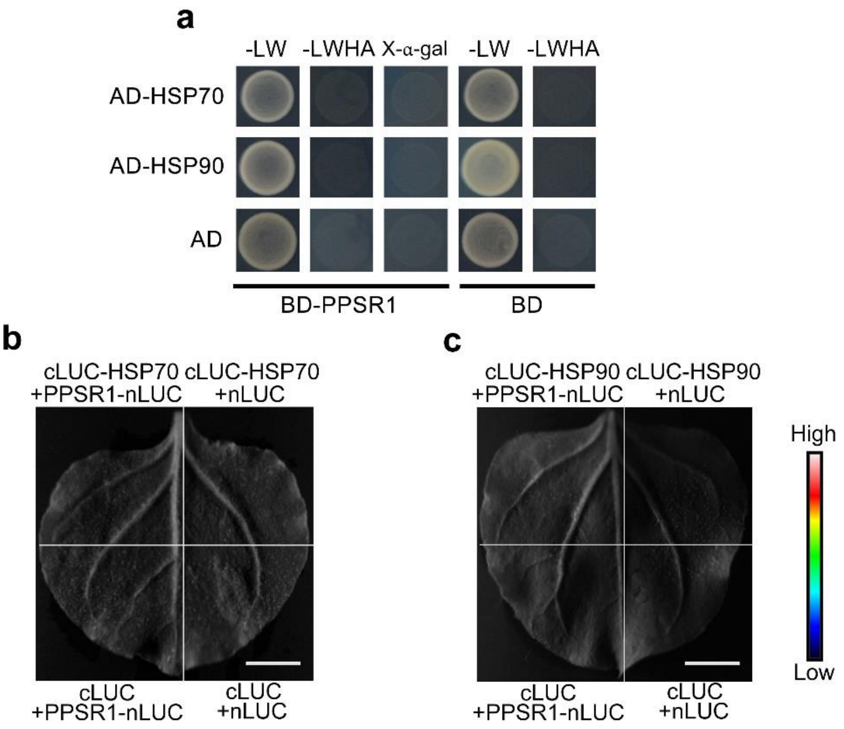

Supplementary Figure 6. PPSR1 does not interact with HSP70 or HSP90.

a, Y2H assay revealing no interactions between PPSR1 and HSP70/HSP90. The PPSR1 fused with the binding domain (BD) of GAL4 (BD-PPSR1) and HSP70/HSP90 fused with the activation domain (AD) of GAL4 (AD-HSP70/AD-HSP90) were co-expressed in yeast. Yeasts expressing BD or AD were used as negative controls. The transformants were selected on SD/-Leu/-Trp (-LW) and SD/-Leu/-Trp/-His/-Ade (-LWHA) with or without X-α-gal. **b** and **c**, LCI assay revealing no interactions between PPSR1 and HSP70/HSP90. The PPSR1 fused with the C-terminus of LUC (cLUC-PPSR1) was co-expressed with HSP70 (**b**) or HSP90 (**c**) fused with the N-terminus of LUC (HSP70-nLUC/HSP90-nLUC) in tobacco (*Nicotiana benthamiana*) leaves. The infected leaves were harvested and observed at 48 h after infiltration. The empty vectors expressing cLUC or nLUC were used as the negative control. Scale bars, 1 cm.

Supplementary Figure 7

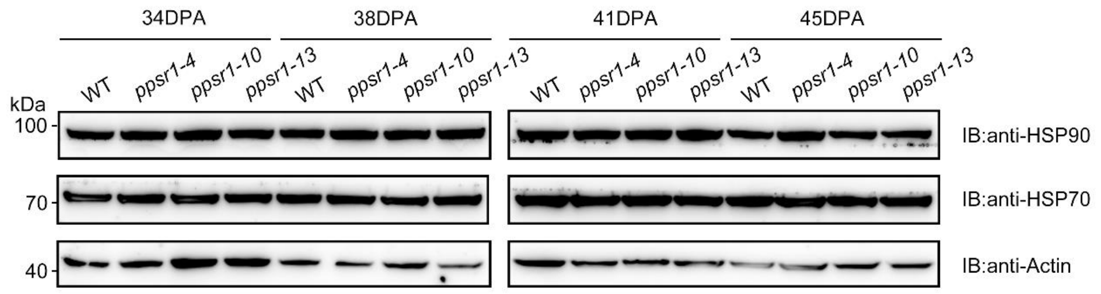

Supplementary Figure 7. The protein levels of HSP70 and HSP90 in tomato fruit during ripening.

Total proteins were extracted from the wild-type (WT) and *ppsr1* tomato fruits at 34, 38, 41, and 45 days post-anthesis (DPA) and submitted to immunoblot using anti-HSP70 and anti-HSP90 antibodies, respectively. Equal loading was confirmed by an anti-actin antibody.

Supplementary Figure 8

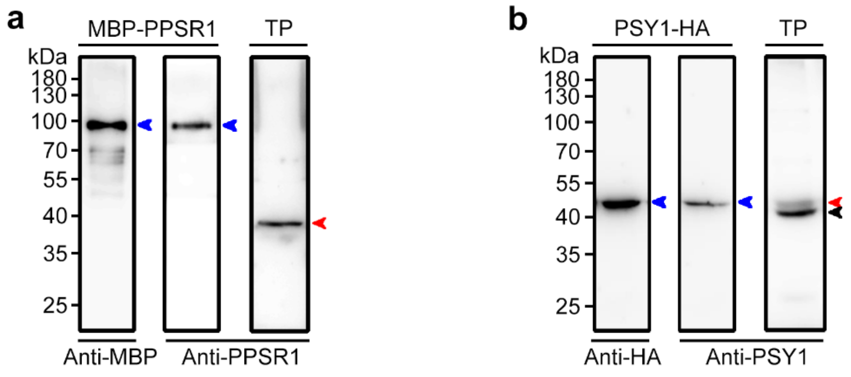

Supplementary Figure 8. Specificity of anti-PPSR1 and anti-PSY1 antibodies.
a, The specificity assay of anti-PPSR1 antibody. Recombinant MBP-PPSR1 protein expressed in *Escherichia coli* and total protein (TP) extracted from wild-type tomato fruit at 38 days post-anthesis were used for immunoblot assay using both anti-MBP and anti-PPSR1 antibodies. The recombinant MBP-PPSR1 protein has a predicted molecular mass of ~90-kDa (indicated by blue arrowhead), and the endogenous PPSR1 protein has a predicted molecular mass of ~38-kDa (indicated by red arrowhead). The experiment was repeated three times and the representative results are shown. **b**, The specificity assay of anti-PSY1 antibody. The PSY1-HA fusion protein expressed in tobacco (*Nicotiana benthamiana*) leaves and total protein (TP) extracted from wild-type tomato fruit at 38 days post-anthesis were used for immunoblot assay using both anti-HA and anti-PSY1 antibodies. The PSY1-HA fusion protein has a predicted molecular mass of ~48-kDa (indicated by blue arrowhead), and the endogenous PSY1 protein has a predicted molecular mass of ~46-kDa (indicated by red arrowhead). Black arrowhead refers to the mature PSY1 protein (~39-kDa). The experiment was repeated three times and the representative results are shown.

Supplementary Figure 9

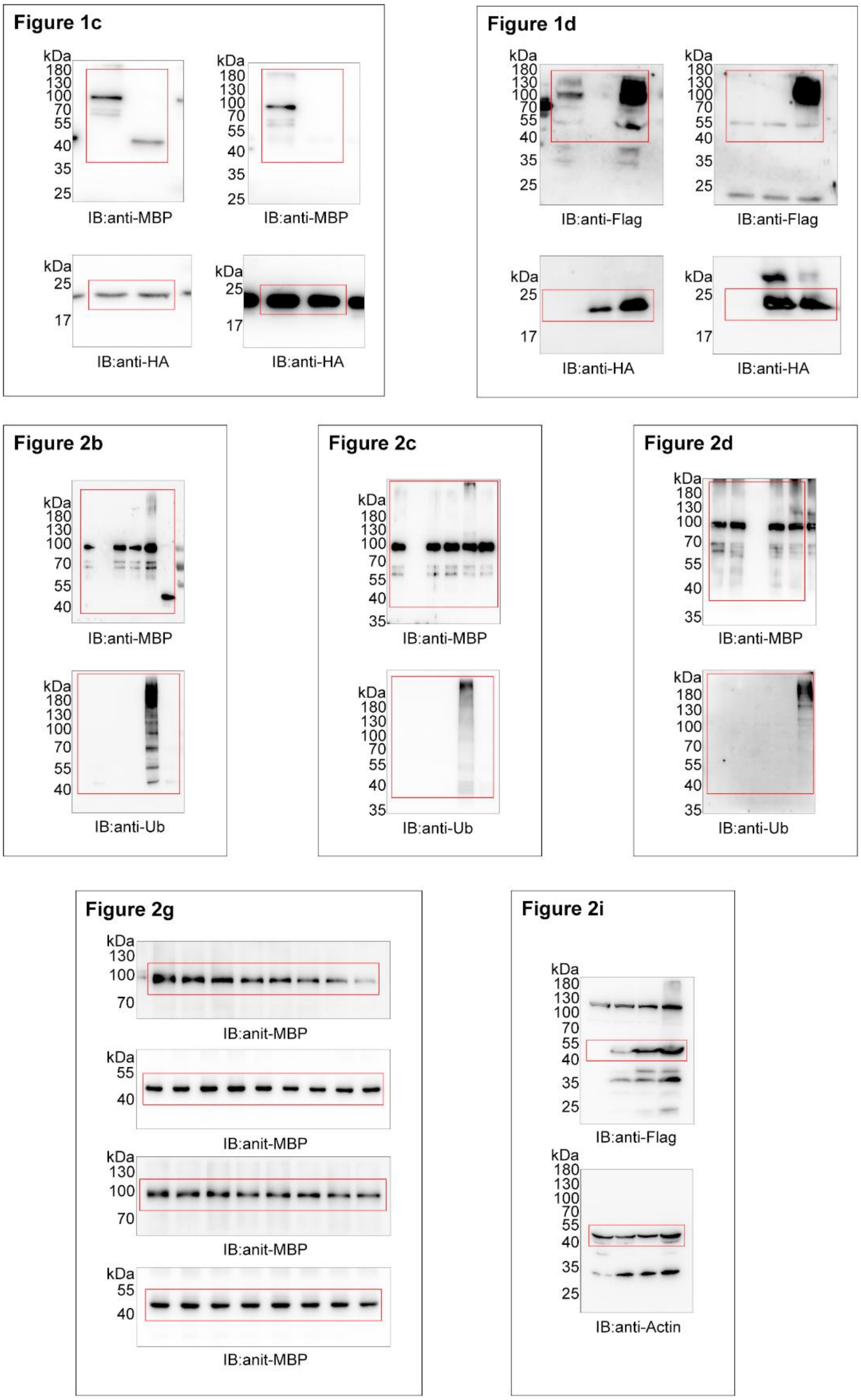

Supplementary Figure 9 (continued)

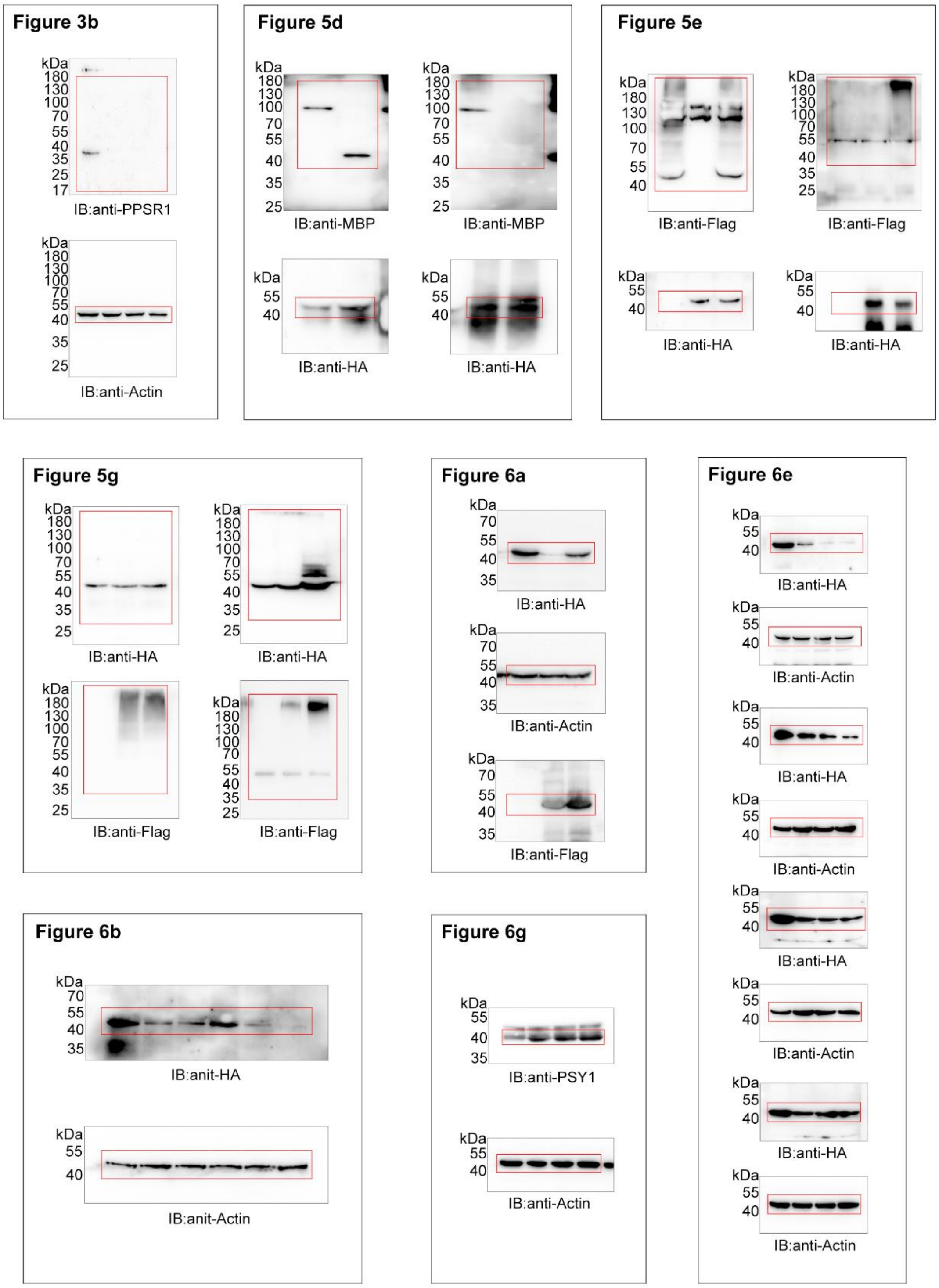

Supplementary Figure 9. Unprocessed original blot images represented in Figure 1–6.

Supplementary Figure 10

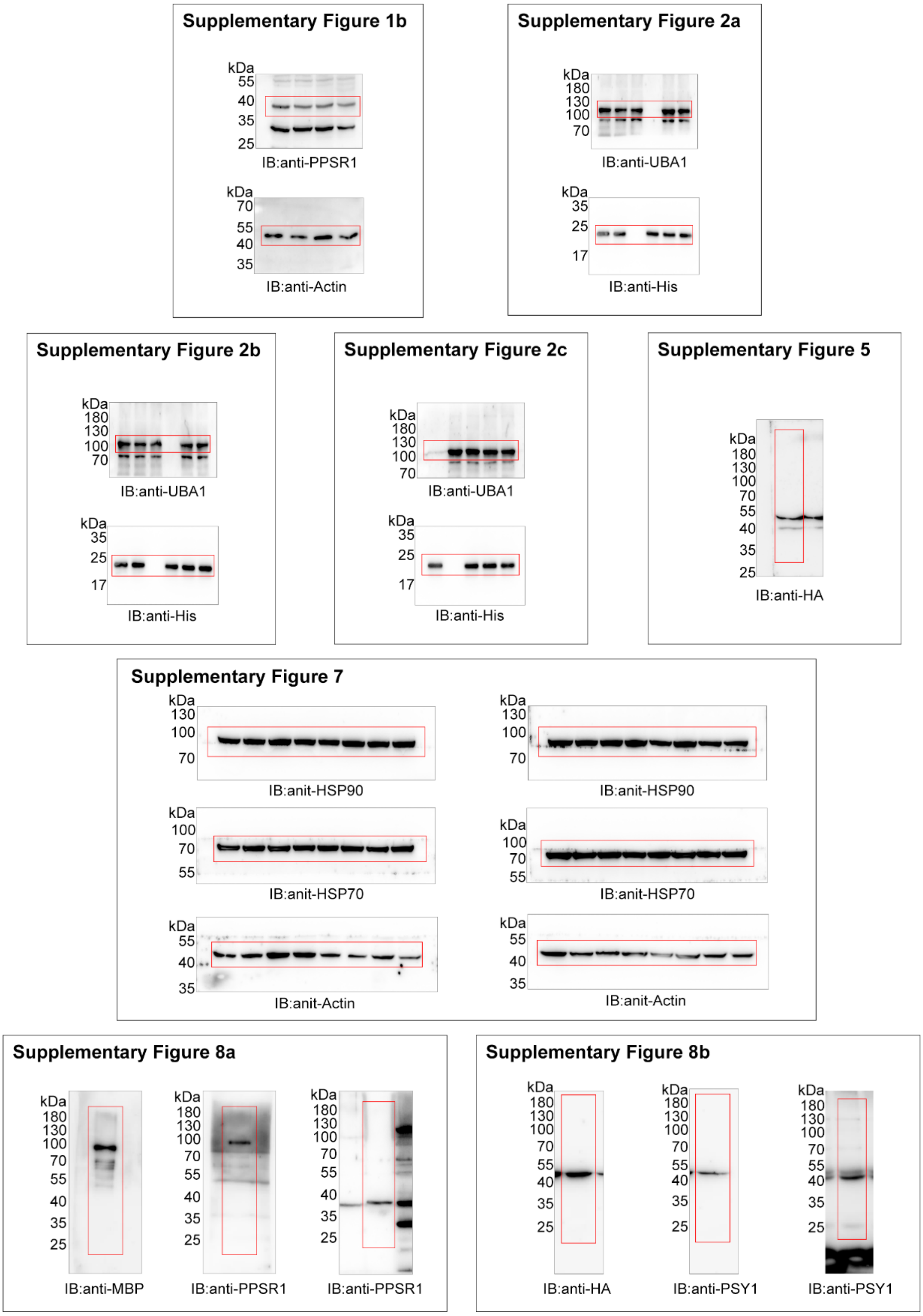

Supplementary Figure 10. Unprocessed original blot images represented in Supplementary Figure 1–8.

Supplementary Figure 11

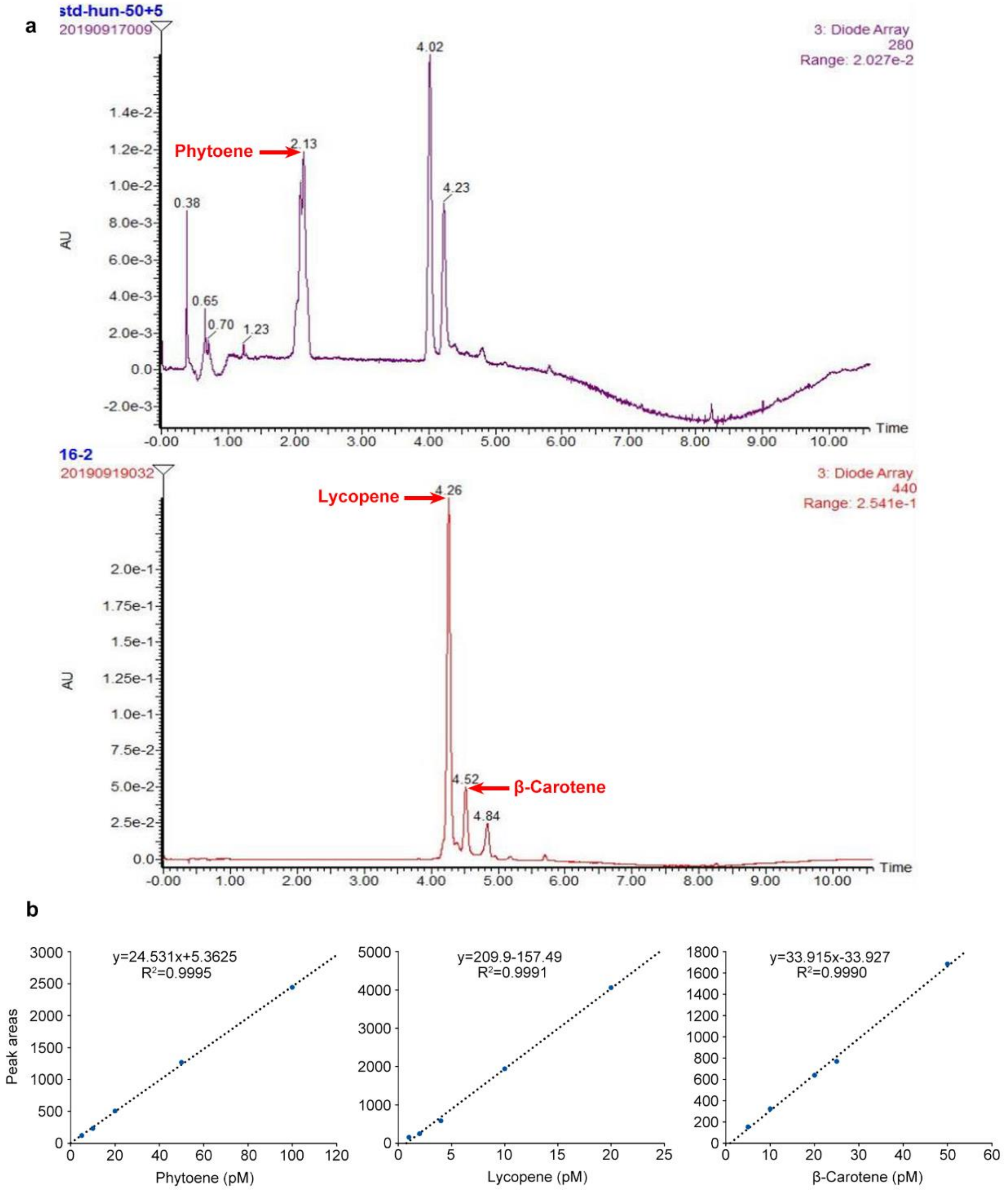

Supplementary Figure 11. Representative liquid chromatogram and the standard curves for phytoene, lycopene, and β-carotene.

a, The amount of phytoene, lycopene, and β-carotene was quantified by their integration areas in the corresponding chromatogram. **b**, The standard curves of phytoene, lycopene, and β-carotene generated by a concentration series of pure commercial reagents.
