# Supplementary material for: Ubiquitination of phytoene synthase 1 precursor modulates carotenoid biosynthesis in tomato
Source: Commun Biol. 2020 Dec 3;3:730. doi: 10.1038/s42003-020-01474-3 (PMC7713427; doi:10.1038/s42003-020-01474-3)
Supplement: Supplementary file 2 — Description of Additional Supplementary Files [file 42003_2020_1474_MOESM2_ESM.pdf]

Description of Additional Supplementary Files

File Name: Supplementary Data 1
Description: Identification of differentially expressed proteins in the ppsr1tomato fruit using iTRAQ-based quantitative proteomic analysis

File Name: Supplementary Data 2
Description: Identification of ubiquitinated peptides that changed abundance in the ppsr1mutant using SWATH-MS approach

File Name: Supplementary Data 3
Description: Primers used in this study

File Name: Supplementary Data 4
Description: Source data for Figure 2, 3, 6and Supplementary Figure 1
