# Supplementary material for: Ubiquitination of phytoene synthase 1 precursor modulates carotenoid biosynthesis in tomato
Source: Commun Biol. 2020 Dec 3;3:730. doi: 10.1038/s42003-020-01474-3 (PMC7713427; doi:10.1038/s42003-020-01474-3)
Supplement: Supplementary file 8 — Reporting Summary [file 42003_2020_1474_MOESM8_ESM.pdf]

nature research

Corresponding author(s): Guozheng Qin

Last updated by author(s): Aug 20, 2020

Reporting Summary

Nature Research wishes to improve the reproducibility of the work that we publish. This form provides structure for consistency and transparency in reporting. For further information on Nature Research policies, see our Editorial Policies and the Editorial Policy Checklist.

Statistics

For all statistical analyses, confirm that the following items are present in the figure legend, table legend, main text, or Methods section.

n/a	Confirmed	
☐	☒	The exact sample size (*n*) for each experimental group/condition, given as a discrete number and unit of measurement
☐	☒	A statement on whether measurements were taken from distinct samples or whether the same sample was measured repeatedly
☐	☒	The statistical test(s) used AND whether they are one- or two-sided<br>*Only common tests should be described solely by name; describe more complex techniques in the Methods section.*
☒	☐	A description of all covariates tested
☒	☐	A description of any assumptions or corrections, such as tests of normality and adjustment for multiple comparisons
☐	☒	A full description of the statistical parameters including central tendency (e.g. means) or other basic estimates (e.g. regression coefficient) AND variation (e.g. standard deviation) or associated estimates of uncertainty (e.g. confidence intervals)
☐	☒	For null hypothesis testing, the test statistic (e.g. *F*, *t*, *r*) with confidence intervals, effect sizes, degrees of freedom and *P* value noted<br>*Give P values as exact values whenever suitable.*
☒	☐	For Bayesian analysis, information on the choice of priors and Markov chain Monte Carlo settings
☒	☐	For hierarchical and complex designs, identification of the appropriate level for tests and full reporting of outcomes
☒	☐	Estimates of effect sizes (e.g. Cohen's *d*, Pearson's *r*), indicating how they were calculated

Our web collection on statistics for biologists contains articles on many of the points above.

Software and code

Policy information about availability of computer code

Data collection	Leica confocal microscope (Leica, DMI600CS) was used to detect the fluorescence from fluorescent fusion protein in subcellular localization observation.<br>Tanon 5200Multi chemiluminescence detector (Tanon, Shanghai, China) was used for capturing the signals of target proteins in immunoblot analysis.<br>Agilent 1290 UPLC system (Agela Technologies) was used to fractionate the peptides in the iTRAQ-based proteomic experiment.<br>NanoLC system (Eksigent, NanoLC–2D Ultra Plus) coupled to a Triple TOF 5600 Plus mass spectrometer (AB SCIEX) was used for the qualitative and quantitative analysis of peptides in the proteomic experiment.<br>ACQUITY UPC2 System (Waters, USA) was used for carotenoid measurement.<br>Step One Plus Real-Time PCR System (Applied Biosystems) was used for detecting the expression pattern of gene.
Data analysis	ImageJ software (https://imagej.nih.gov/ij/index.html) was used to quantify the target protein level in immunoblot assays.<br>GraphPad Prism version 8.0 (https://www.graphpad.com/scientific-software/prism) and Excel 2010 (https://products.office.com/en-us/office-2010) was used for statistical analysis.<br>ProteinPilotTM 4.5 (AB SCIEX) and Pro GroupTM algorithm (AB SCIEX) softwares were used for the protein identification and relative quantification in mass spectra data.<br>ProteinPilotTM 4.5 (AB SCIEX) and Peakview (AB SCIEX) softwares were used for the identification and quantification of ubiquitinated peptides.<br>MarkerView (AB SCIEX) was used for visualizing the ion transitions from the ubiquitinated peptides.

For manuscripts utilizing custom algorithms or software that are central to the research but not yet described in published literature, software must be made available to editors and reviewers. We strongly encourage code deposition in a community repository (e.g. GitHub). See the Nature Research guidelines for submitting code & software for further information.

Data

Policy information about availability of data

All manuscripts must include a data availability statement. This statement should provide the following information, where applicable:

- Accession codes, unique identifiers, or web links for publicly available datasets
- A list of figures that have associated raw data
- A description of any restrictions on data availability

All data that support the findings of this study are available from the corresponding author upon request. The mass spectrometry proteomics data for iTRAQ and ubiquitinated peptide identification have been deposited in the PRIDE archive (Nos. PXD018731 and PXD018707, respectively; https://www.ebi.ac.uk/pride/archive).

Field-specific reporting

Please select the one below that is the best fit for your research. If you are not sure, read the appropriate sections before making your selection.

- [x] Life sciences
- [ ] Behavioural & social sciences
- [ ] Ecological, evolutionary & environmental sciences

For a reference copy of the document with all sections, see nature.com/documents/nr-reporting-summary-flat.pdf

Life sciences study design

All studies must disclose on these points even when the disclosure is negative.

Sample size	The detailed sample size is indicated in the Methods. The sample size in this study mainly depends on the previous reports or experiences, which are based on representativeness and statistical significance of the results in the experiments.
Data exclusions	No data were excluded from the analysis.
Replication	The number of replications is indicated in the Figure legends and Methods. For the proteomic experiment shown in Figure 3 and Figure 4, two independent biological replicates were performed. The other major experiments were conducted at least three times. Similar results were obtained.
Randomization	The tomato (Solanum lycopersicum) plants of different genotypes were randomly grown in the same greenhouse to ensure as equal growth conditions as possible. Fruit samples were randomly harvested from different plants in the same experimental group. The tobacco (Nicotiana benthamiana) plants grown under the same culture conditions (growth room) were randomly chosen for the transient expression of protein in the study.
Blinding	Experiments were not blinding. Data collection was carried out according to the genotype of plant.

Reporting for specific materials, systems and methods

We require information from authors about some types of materials, experimental systems and methods used in many studies. Here, indicate whether each material, system or method listed is relevant to your study. If you are not sure if a list item applies to your research, read the appropriate section before selecting a response.

Materials & experimental systems

n/a	Involved in the study	
☐	☒	Antibodies
☒	☐	Eukaryotic cell lines
☒	☐	Palaeontology and archaeology
☒	☐	Animals and other organisms
☒	☐	Human research participants
☒	☐	Clinical data
☒	☐	Dual use research of concern

Methods

n/a	Involved in the study	
☒	☐	ChIP-seq
☒	☐	Flow cytometry
☒	☐	MRI-based neuroimaging

Antibodies

Antibodies used	Anti-Flag monoclonal antibody in mouse (MBL life science, Catalog: M185-3S, 1:10000 dilution, more information at https://ruo.mbl.co.jp/bio/e/index.html).<br>Anti-HA monoclonal antibody in mouse (Abmart, Catalog: M20003L, 1:5000 dilution, more information at https://www.ab-mart.com.cn).<br>Anti-Actin monoclonal antibody in mouse (Abmart, Catalog: M20009M, 1:5000 dilution, more information at https://www.ab-mart.com.cn).<br>Anti-MBP monoclonal antibody in mouse (Beijing Protein Innovation, Catalog: AbM59007-3-PU, 1:5000 dilution, more information at

http://www.proteomics.org.cn).
Anti-Ubiquitin (P4D1) monoclonal antibody in mouse (Santa Cruz Biotechnology, Catalog: sc-8017, 1:2000 dilution, more information at https://www.scbt.com).
Anti-HSP70 monoclonal antibody in mouse (Abmart, Catalog: M51012M, 1:5000 dilution, more information at https://www.ab-mart.com.cn).
Anti-HSP90 monoclonal antibody in mouse (Abmart, Catalog: M51057M, 1:5000 dilution, more information at https://www.ab-mart.com.cn).
Anti-His (2A8) monoclonal antibody in mouse (Abmart, Catalog: M20001L, 1:5000 dilution, more information at https://www.ab-mart.com.cn).
Anti-UBA1 polyclonal antibody in rabbit (BOSTER, Catalog: PB0950, 1:5000 dilution, more information at http://www.boster.com.cn).
HRP-conjugated anti-mouse (Catalog: M21001L) and anti-rabbit (Catalog: M21002S) secondary antibodies (Abmart, 1:5000 dilution, more information at https://www.ab-mart.com.cn).
Anti-PPSR1 and anti-PSY1 polyclonal antibodies in rabbit were custom-made in the Abmart Shanghai Co., Ltd (Shanghai, China), the detailed information described in the Methods.

Validation

Validation statement for anti-Flag antibody (MBL life science, Catalog: M185-3S) can be found at the product website. <https://ruo.mbl.co.jp/bio/e/dtl/A/?pcd=M185-3S>
Validation statement for anti-HA antibody (Abmart, Catalog: M20003L) can be found at the product website. <http://www.ab-mart.com.cn/page.aspx?node=60&id=963>
Validation statement for anti-Actin antibody (Abmart, Catalog: M20009M) can be found at the product website. <http://www.ab-mart.com.cn/page.aspx?node=59&id=985>
Validation statement for anti-MBP antibody (Beijing Protein Innovation, Catalog: AbM59007-3-PU) can be found at the product website. <http://www.proteomics.org.cn/product/189.html>
Validation statement for anti-Ubiquitin (P4D1) antibody (Santa Cruz Biotechnology, Catalog: sc-8017) can be found at the product website. <https://www.scbt.com/zh/p/ubiquitin-antibody-p4d1?requestFrom=search>
Validation statement for anti-HSP70 antibody (Abmart, Catalog: M51012M) can be found at the product website. <http://www.ab-mart.com.cn/page.aspx?node=67&id=28>
Validation statement for anti-HSP90 antibody (Abmart, Catalog: M51057M) can be found at the product website. <http://www.ab-mart.com.cn/page.aspx?node=67&id=1078>
Validation statement for anti-His (2A8) antibody (Abmart, Catalog: M20001L) can be found at the product website. <http://www.ab-mart.com.cn/page.aspx?node=60&id=959>
Validation statement for anti-UBA1 antibody (BOSTER, Catalog: PB0950) can be found at the product website. <http://www.boster.com.cn/product/anti-uba1-antibody_pb0950.html>
Validations for anti-PPSR1 and anti-PSY1 polyclonal antibodies are conducted and showed in the Supplementary Figure 8.
